# Systemic therapy of non-colorectal gastrointestinal malignancies in the elderly

**DOI:** 10.7497/j.issn.2095-3941.2015.0078

**Published:** 2015-12

**Authors:** Avni M. Desai, Stuart M. Lichtman

**Affiliations:** Department of Medicine, Memorial Sloan Kettering Cancer Center, New York, NY 11725, USA

**Keywords:** Pancreatic cancer, esophageal cancer, biliary cancer, hepatocellular carcinoma (HCC), chemotherapy, elderly, geriatrics, gastric cancer

## Abstract

In the coming years life expectancy is expected to increase and with this the percentage of the population above age 65 will grow. Patients above 65 make up more than two thirds of those currently diagnosed with gastrointestinal malignancies. Available evidence based medicine does not focus on the average patient, above the age 70, encountered in every day practice. Most guidelines and clinical trials are not designed to take into account the special considerations needed when treating the elderly such as functional status, comorbidities, polypharmacy, life expectancy, and social support. The majority of available data is based on retrospective reviews or subset analyses of larger studies where the elderly represent a fraction of the studied population. This review focuses on the toxicities and tolerability of current standard therapies for non-colorectal gastrointestinal malignancies, including gastroesophageal, pancreatic, bile duct and hepatocellular cancers in the elderly. With careful patient selection and geriatric assessment the elderly can safely benefit from standard therapies offered to younger patients.

## Introduction

More than two thirds of gastrointestinal cancers are diagnosed in patients older than age 65 (**Table 1**). In coming years the percentage of the population over age 65 is only expected to increase as life expectancy increases due to the aging of the population and control of comorbidity^1^. Evidence based medicine is lacking for the elderly as many studies do not include many patients above the age of 70^2^. Clinical trials are not specifically designed to evaluate this age group, limiting our understanding of the toxicities and benefits of cancer therapies in the elderly. Patients aged 65 and over represent only 22%-32% of patients enrolled in clinical trials, while they represent 63% of the population with cancer^3^^-^^7^.

**Table 1:** Median age at diagnosis and death of non-colorectal gastrointestinal malignancies

Site	Median age at diagnosis	Median age at death	5-year survival (%)
Pancreas	71	73	7.2
Esophagus	67	69	17.9
Stomach	69	72	29.3
Liver and intrahepatic bile duct	63	67	17.2
Small intestine	65	72	65.5

Ref: seer.cancer.gov (accessed 9/14/2015).

There are no clear guidelines to treat elderly patients and they are often not offered therapies that are known to improve overall survival (OS). Concerns regarding ability to tolerate therapy, comorbidities and poor social support hinder accrual of older patients to clinical trials^8^. Small studies and subset analyses have shown older patients can tolerate chemotherapy at similar dose intensity as younger patients without a significant impact on quality of life^9^.

Areas of particular concern in the aging population include comorbidities, polypharmacy, life expectancy, and social support. Physiologic age rather than chronologic age predicts toxicity associated with therapy. In particular, functional capacity, which does not correlate with Karnofsky performance score (KPS) or Eastern Cooperative Oncology Group (ECOG) performance status and comorbidity, is a useful predictor of the toxicity and benefit of systemic chemotherapy, as well as survival, morbidity and mortality in elderly cancer patients^10^. Patients above the age of 80 are more likely to be functionally dependent, and incapable of independent living. They often require help with instrumental activities of daily living (IADLs) as well as activities of daily living (ADLs). IADLs consist of ability to prepare meals, go shopping, utilize transportation, manage finances, and phone use. ADLs include feeding, bathing, and dressing. The survival of the elderly is often reliant on social support and caregiver assistance^11^^-^^13^.

The elderly are a heterogeneous population making incorporation of assessments such as comprehensive geriatric assessment (CGA), which includes ADL/IADLs evaluation, Mini-Mental Status Examination (MMSE) and geriatric depression scale (GDS) important. Assessments such as frailty screens and CGA can aid in preventing both over and under treatment of patients of a certain age^14^. In a study of patients above the age of 70, with the observational group receiving standard chemotherapy and the intervention group receiving a geriatrician led CGA, the group undergoing CGA were more likely to finish planned chemotherapy with less dose modifications^15^. Life expectancy predictors can also be useful in determining which elderly patients may derive benefit from curative or palliative therapy^16^^-^^19^.

Other precautions in treatment must be taken when treating older patients with chemotherapy due to decrease in glomerular filtration rate, impaired ability to repair mucosal barriers and decreased hematopoietic reserve. Toxicities include cardiotoxicity, mucocitis and neuropathy maybe more debilitating in older patients^20^^-^^23^. This review focuses on the toxicities and tolerability of current standard therapies for non-colorectal gastrointestinal malignancies, including gastroesophageal, pancreatic, bile duct and hepatocellular cancers in the elderly. Unfortunately, there are no large prospective studies in the elderly patient population. This highlights that these trials are needed so that clinicians can make evidenced based decisions^2^.

## Pancreatic cancer

The 5-year OS for pancreatic cancer is less than 5%, and the majority of diagnosed patients are above the age of 65. Pancreatic cancer is the seventh most common cause of cancer related deaths in both males and females worldwide based on data collected by the World Health Organization (WHO). In developed countries it is the fourth most common cause of cancer related deaths. Statistics are based on GLOBOCAN estimates from the International Agency for Research on Cancer (IARC)^24^^,^^25^.

### Adjuvant therapy

Older patients are more likely to present at an early stage and surgery is the only curative option. Surgical resection with pancreatoduodenectomy is safe in the elderly. In 138 patients at Memorial Sloan Kettering Cancer Center, age >70, operative mortality and complication rate was similar to that of younger patients. OS was thought to be slightly lower due to age related comorbidities^26^.

Following pancreatic cancer two agents have shown benefit in improving OS in the adjuvant setting, 5-fluorouracil (5-FU) and gemcitabine. The European Study Group for Pancreatic Cancer (ESPAC-1) trial showed an increase in OS of 19.7 *vs.* 14 months with 5-FU, compared to no chemotherapy (HR =0.66; *P*=0.0005)^27^. Gemcitabine was found to improve OS and progression free survival (PFS) compared to no chemotherapy, median OS 22.8 *vs.* 20.2 months (*P*=0.005), in the CONKO-1 study^28^. Subsequently gemcitabine and 5-FU/leucovorin (LV) groups showed similar OSs of 23 and 23.6 months in the ESPAC-3 trial^29^. Gemcitabine is recommended in elderly patients, as there were less adverse events than in the 5-FU arm.

The average age of patients with pancreatic cancer in the community does not reflect that of patients in larger phase III clinical trials. The elderly are underrepresented, with ESPAC-1 having a median age of 61 and Charité Onkologie (CONKO-001) having a median age of 61.

A smaller Australian study demonstrated older patients (aged >70) were less likely to receive adjuvant chemotherapy (51.5% *vs.* 29.8%; *P*<0.0001). Patients above the age of 70 had an exceptionally poor outcome when adjuvant therapy was not delivered (median survival =13.1 months; HR =1.89; 95% CI, 1.27-2.78; *P*=0.002)^30^. A population based study showed patients of older age were less likely to receive adjuvant therapy^31^. Selected elderly patients derive similar benefit from adjuvant therapies; however they were less likely to receive them^32^. In a retrospective review of resected patients, there was no difference in outcomes when patients over 70 years were compared to younger^33^.

### Chemoradiation in the adjuvant and locally advanced setting

Multiple trials have shown the use of chemoradiation in the adjuvant setting has been of conflicting benefit in improvement of OS. In a series of patients >75 receiving chemoradiation in the locally advanced or adjuvant setting, a similar OS to younger patients receiving similar therapy was noted. In a retrospective analysis of patients who underwent pancreatoduodenectomy at Johns Hopkins Hospital, adjuvant therapy in elderly patients ≥75 had a protective effect with respect to 2-year survival [relative risk (RR) =0.58; *P*=0.044), but not 5-year survival (RR =0.80; *P*=0.258). For patients less than age 75, adjuvant chemoradiation was significantly associated with 2- and 5-year survival^34^.

### Metastatic disease

Gemcitabine has been established as the superior to 5-FU in patients with metastatic disease with median OS of 5.6 *vs.* 4.4 months. The 1-year OS was 18% *vs.* 2%. Significant clinical improvement was noted in 23.8% of patients with gemcitabine *vs.* 4.8% of patients treated with 5-FU^35^. A retrospective study showed that 59% of patients ≥70 were able to receive full does of gemcitabine 100 mg/m^2^ weekly for 3 weeks of a 4-week cycle. Patients >70 had similar toxicities, OS and benefit compared to those below the age of 70 in a pooled analysis of 7 prospective phase II and III clinical trials^36^. PFS and OS were the same as for younger historical patients. Aside from the addition of erlotinib and nab paclitaxel to gemcitabine, other combinations of chemotherapy with gemcitabine have not been shown to improve OS. The addition of erlotinib in the elderly is not routinely recommended as it improves OS by 6.24 *vs.* 5.91 months^37^. Nab paclitaxel in combination with gemcitabine improved OS by 1.8 months over gemcitabine alone. The median age of patients was 62, with at least 10% older than age 75 and 8% with an ECOG PS 2 ^38^. FOLFIRINOX (5-FU, oxaliplatin, irinotecan, leucovorin) has been shown to improve OS by 4.3 months over gemcitabine; however patients above the age of 75 were excluded. The significantly higher incidence of grade 3 or 4 adverse events with FOLFIRIOX *vs.* gemcitabine, including neutropenia and neuropathy, may make FOLFIRINOX less tolerable in the frailer elderly population^39^.

A German retrospective analysis of 53 patients ≥70, 81% of whom received gemcitabine chemotherapy showed similar survivals to those of younger age. Second line therapy appeared to improve survival relative to best supportive care alone. The 5-FU oxaliplatin combinations have shown benefit in this setting^40^.

## Gastroesophageal cancers

### Gastric cancer

The majority of gastroesophageal cancers are diagnosed in patients older than age 65. Based on statistics from data collected by the WHO and GLOBOCAN estimates from the IARC, gastric cancer is the third most common cause of cancer related deaths in males and the fifth most common cause of cancer related deaths in females worldwide. In developing countries it is the third most common cause of cancer related deaths in males and the fourth most common cause of cancer related deaths in females. An estimated 951,600 new stomach cancer cases and 723,100 deaths occurred in 2012. Incidence varies by country, remaining high in countries in Eastern Asia, South America and Central and Eastern Europe. Gastric cancer is now less common in North America and Western European countries^25^^,^^41^.

#### Perioperative therapy

The addition of chemotherapy improves OS compared to curative surgery alone. The Medical Research Council Adjuvant Gastric Infusional Chemotherapy (MAGIC) trial showed perioperative chemotherapy with epirubicin, cisplatin and 5-FU (ECF) improved 5-year survival from 23% to 36.3% compared to surgery alone. About 20% of patients were ≥70. Survival benefit was similar in those older than 70 compared to those who were younger. Only half of the patients were able to receive the adjuvant portion of chemotherapy^42^. Given concerns for cardiotoxicity with epirubicin and similar survival benefits of 5-FU and platinum regimens compared to regimens containing 5-FU, platinum and epirubicin, epirubicin is not often used in the elderly. Based on REAL-2 data showing less toxicity and increased OS with epirubicin, oxaliplatin, capecitabine (EOX) over ECF, oxaliplatin is preferred over cisplatin in combination with 5-FU or capecitabine.

#### Adjuvant therapy

Adjuvant therapy for gastric cancer was shown to improve OS in a meta-analysis^43^. Most benefit is derived from 5-FU-based chemotherapy and the benefits were independent on age. Based on the intergroup trial 0116, adjuvant 5-FU followed by chemoradiation and additional 5-FU is used in the United States due to significant improvement in OS and relapse free survival. Of note greater than 50% of those patients had less than a D1 resection. About 54% underwent a D0 resection and 36% underwent a D1 resection. 10% underwent D2 resection, which is standard in Japan^44^. D2 lymphadenectomy, involving a more extensive lymph node dissection compared to D1 resection of lymph nodes close to the primary tumor, has been associated with decreased locoregional recurrence and gastric cancer related mortality relative to D1 resection. These results have differed between Western and Eastern countries^45^^-^^48^.

The use of adjuvant fluoropyrimidine was also supported with the S-1 trial from Japan showing 80.1% *vs.* 70.1% 3-year OS benefit. S-1 therapy is standard for 1 year following D2 resection versus surgery alone in Japan based on these findings. Of patients treated on the S1 arm, 25.9% of patients were of ages >70-80 years. The benefit of improved OS was present across all age groups^49^. The capecitabine and oxaliplatin adjuvant study in stomach cancer (CLASSIC) trial evaluated adjuvant capecitabine and oxaliplatin, demonstrating 74% DFS with chemotherapy versus 59% in the D2 resection arm alone^50^.

### Esophageal cancer

An estimated 455,800 new esophageal cancer cases and 400,200 cancer deaths occurred in 2012 based on data collected by the WHO and GLOBOCAN estimates from the IARC. The highest incidence of esophageal cancer is in Eastern Asia, Eastern and Southern Africa, with the lowest in Western Africa^25^.

#### Preoperative chemoradiation

Preoperative therapy for locally advanced esophageal adenocarcinoma has been studied in a number of randomized trials prior to curative surgical resection. Therapy in the preoperative setting is often better tolerated than adjuvant therapy and the elderly benefit with a slight increase in toxicity^51^. Patients above the age of 70 had similar 3-year OS to those who were younger. Carboplatin and paclitaxel has been utilized as a radiosensitizer as per the Chemoradiotherapy for Oesophageal Cancer Followed by Surgery Study (CROSS) trial. Although the median age in the trial was 60, patients up to age 79 were enrolled^52^. A series of patients at the University of Texas MD Anderson Cancer Center receiving preoperative chemoradiation were found to have similar perioperative mortality and post operative complication rate in patients greater than age 70 compared to those less than age 70^51^. Although surgical resection is the mainstay of curative therapy, definitive chemoradiation is utilized in patients with squamous cell carcinoma rather than adenocarcinoma with complete clinical response and in patients who are poor surgical candidates.

#### Metastatic disease

Patients above the age of 65 are not well represented in trials for metastatic gastroesophageal cancer. The median age of patients the V325 trial showing improved OS of 9.2 *vs.* 8.6 months for docetaxel, cisplatin and 5-FU (DCF) *vs.* 5-FU and cisplatin (EF) alone was 55 years. Patients aged 65 and above comprised 24% of enrolled patients. Higher incidence of toxicity did not affect clinical benefit and quality of life. Grade 3 and 4 infection related to treatment occurred more often in those aged 65 or older in DCF (20%) *vs.* CF (9%). Febrile neutropenia occurred in 29% of patients with DCF^53^.

Non-inferiority in OS of EOX compared to ECF, ECX and EOX was established by the REAL-2 [randomised study of ECF for advanced or locally advanced esophagogastric cancer (OG)] trial. EOX was shown to have increased OS of 11.2 *vs.* 9.9 months with ECF with less toxicity. The median age in this trial was between age 61 and 65 in the different arms^54^. Given concerns for cardiotoxicity with epirubicin and similar survival benefits of 5-FU and platinum regimens compared to regimens containing 5-FU, platinum and epirubicin, epirubicin is not often used in the elderly.

In patients with human epithelial growth factor receptor (HER2) amplified gastric and gastroesophageal junction tumors, the addition of trastuzumab to 5-FU and cisplatin showed a survival advantage of 13.8 *vs.* 11 months for chemotherapy alone in the trastuzumab for gastric cancer (ToGA) trial. A prespecified sub-group of patients by age demonstrated patients above age 60 also had a significant benefit in OS. Around 5% of patients developed asymptomatic decrease in EF to <50%^55^.

Small studies and pooled analysis for patients aged ≥70 showed no change in grade III/IV toxicities, OS and response rates. A German retrospective analysis of 55 patients ≥70 years, the majority of whom received a combination of two cytotoxic medications showed survival similar to current phase III trial results for advanced gastroesophageal cancer that included patients with a younger median age. PFS and OS were 5.8 and 9.5 months. Second line therapy appeared to have benefit compared to best supportive care. Patients above the age of 75 had similar OS. Those with ECOG PS ≤2 had worse outcomes^40^.

## Hepatocellular carcinoma (HCC)

HCC is the second most common cause of cancer related death worldwide in men. Increased rates are found in less developed countries of Asia, Northern and Western Africa. More developed countries have lower incidence^25^. The average age of patients with HCC continues to increase worldwide with a median age of diagnosis of 63 years in the United States. Multiple retrospective reviews have shown similar OS in patients above the age of 70 compared to those less than 70. As survival has been shown to be dependent on stage of HCC rather than age, elderly with good functional status should be considered for all standard treatment options, including local therapies such as transcatheter arterial chemoembolization (TACE) or resection as appropriate^56^^-^^58^.

### Advanced disease

For advanced or metastatic HCC not amenable to local therapy, the standard of care is sorafenib, an oral multikinase inhibitor. The phase III Sorafenib Hepatocellular Carcinoma Assessment Randomized Protocol (SHARP) trial, with a median age of 64.9 years, showed sorafenib increased OS by 3 months compared to placebo^59^. Subsequently, a series of patients, with a median age of 76, had a similar median OS of 10 months compared to 10.7 months in the SHARP trial^60^. Sorafenib also appears to be well tolerated in this series of elderly patients, with similar treatment related adverse effects noted as in the SHARP trial^60^.

## Biliary tract cancers

The incidence of biliary tract cancer, including cholangiocarcinoma and gallbladder cancer, increases with age. While biliary tract carcinoma is uncommon in Western countries there is increased prevalence in other areas of the world, such as Southeast Asia. In a Japanese review, curative resection had similar mortality and survival rates for patients over the age of 75 compared to those younger than 75.

### Metastatic disease

For advanced disease the phase III advanced biliary cancer (ABC-02) trial demonstrated an increase in median survival of 11.7 *vs.* 8.1 months with cisplatin and gemcitabine compared to the gemcitabine alone group. While the median age was only 63.9 years, the study included patients up to age 81.9^61^. A Japanese retrospective analysis of patients treated with palliative chemotherapy for biliary tract cancers showed similar OS of 10.4 *vs.* 11.5 months in patients ≥75 compared to those <75 years. The frequency of adverse effects was similar between both age groups^62^. Another retrospective analysis including biliary tract cancers as well as pancreatic cancer showed similar adverse events, dose intensities and OS in the elderly compared to younger patients who received systemic gemcitabine^63^. A small retrospective review of 20 patients with a median age of 74 showed manageable toxicities and comparable OS to younger patients^64^. Systemic therapy with gemcitabine alone rather than combination chemotherapy with gemcitabine and a platinum agent may be more appropriate for elderly with decreased functional status and multiple comorbidities due to concerns of increased toxicity with cisplatin.

## Conclusion

As the population ages and the number of patients over the age of 65 grow, it is increasingly important to understand that the elderly are a heterogeneous group. Incorporating an increasing percentage of patients above 65 into clinical trials, reflecting the average patient population, will enhance our understanding of the benefits and challenges in selecting both appropriate therapies as well as the appropriate patients for treatment in an elderly population.

While awaiting increased evidence based data, patients with good functional status and adequate life expectancy, exceeding the expected survival from the diagnosed malignancy, should be offered standard chemotherapy without dose reduction. For those with multiple comorbidities, poor functional status and decreased life expectancy, best supportive care or therapy modifications are appropriate. Future studies including the elderly with a range of functional status are needed to help guide therapy in the population most affected by gastrointestinal malignancy^65^^,^^66^.
